# Preservation of Medical Records

**Published:** 1909-10

**Authors:** Charles A. L. Reed, Otto Juettner, A. G. Drury

**Affiliations:** 710 W. Eighth St., Cincinnati, O.; 710 W. Eighth St., Cincinnati, O.; 710 W. Eighth St., Cincinnati, O.


### CORRESPONDENCE.

### Preservation of Medical Records

**The Western Association for the Preservation of Medical Records made an Appeal to the Medical Profession of the West and South—It is Desired to Preserve Historical and Biographical Records—The Lloyd Library, Cincinnati, becomes the Custodian.**

*Editor Buffalo Medical Journal:*

SIR—Up to the present time there has not been a concerted effort made to collect and preserve historical data in regard to the origin, evolution and personnel of our profession in this part of our country. The result of this delinquency has been the total loss of much material that should have been preserved, especially pertaining to medical schools and societies, and biographical matter in connection with the practitioners and teachers of medicine of by-gone-days. A good deal of material of this character is still obtainable if a systematic effort is made to locate and preserve it. It is in the possession of individuals, families and private libraries and will eventually be lost. The Western Association for the Preservation of Medical Records was organised in May, 1909, for the purpose of collecting the historical and biographical records of the profession of the west and south. We wish to preserve anything and everything pertaining to western medicine and medical men, and are anxious to enlist the active help and support of every member of the profession who is in sympathy with our aims. We want every one to become associated and identified with the work of our association. There are no fees or obligations of any kind. We have made arrangements with the Lloyd Library, Cincinnati, O., for the proper housing of the material collected. The latter will be systematically arranged, catalogued and properly preserved so that it can be made available for research work. We are particularly anxious to obtain (1) medical journals published in the west and south prior to 1880; (2) medical books and pamphlets written or published in the west and south; (3) manuscripts and autographs of early physicians; (4) old diplomas and other documents of a medical character; (5) proceedings of medical societies; (6) reports of hospitals and other medical institutions; (7) catalogues and announcements of western and southern colleges of all "schools;" (8) biographies and portraits of western physicians; (9) information and material of any kind pertaining to medicine and medical men and affairs in the west; (10) curios of a medico-historical character.

All contributions should be sent in care of the librarian. In view of the fact that we are performing a labor of love and have no funds, our friends and associates will readily understand why all contributions sent by express or freight should be prepaid so that no expense may accrue to the association. The necessary expenses of the association are at present being met by voluntary contributions of its organisers.

May we not count upon your active help and support? We would like to hear from every member of the profession who is interested in the proposed work.

Charles A. L. Reed, M.D., Chairman,
Otto Juettner, M.D., Secretary.

A. G. Drury, M.D., Librarian,
710 W. Eighth St., Cincinnati, O.

